# Design and testing of a synthetic biology framework for genetic engineering of *Corynebacterium glutamicum*

**DOI:** 10.1186/1475-2859-11-147

**Published:** 2012-11-07

**Authors:** Pablo Ravasi, Salvador Peiru, Hugo Gramajo, Hugo G Menzella

**Affiliations:** 1 Genetic Engineering & Fermentation Technology. Instituto de Biología Celular y Molecular de Rosario-CONICET. Facultad de Ciencias Bioquímicas y Farmacéuticas, Universidad Nacional de Rosario, Suipacha 531, Rosario, 2000, República Argentina; 2 Geneg SRL, Cuba 4710, Buenos Aires, Argentina

**Keywords:** Synthetic biology, Metabolic engineering, *Corynebacterium glutamicum*

## Abstract

**Background:**

Synthetic biology approaches can make a significant contribution to the advance of metabolic engineering by reducing the development time of recombinant organisms. However, most of synthetic biology tools have been developed for *Escherichia coli.* Here we provide a platform for rapid engineering of *C. glutamicum*, a microorganism of great industrial interest. This bacteria, used for decades for the fermentative production of amino acids, has recently been developed as a host for the production of several economically important compounds including metabolites and recombinant proteins because of its higher capacity of secretion compared to traditional bacterial hosts like *E. coli*. Thus, the development of modern molecular platforms may significantly contribute to establish *C. glutamicum* as a robust and versatile microbial factory.

**Results:**

A plasmid based platform named pTGR was created where all the genetic components are flanked by unique restriction sites to both facilitate the evaluation of regulatory sequences and the assembly of constructs for the expression of multiple genes. The approach was validated by using reporter genes to test promoters, ribosome binding sites, and for the assembly of dual gene operons and gene clusters containing two transcriptional units. Combinatorial assembly of promoter (*tac*, *cspB* and *sod*) and RBS (*lacZ*, *cspB* and *sod*) elements with different strengths conferred clear differential gene expression of two reporter genes, eGFP and mCherry, thus allowing transcriptional “fine-tuning”of multiple genes. In addition, the platform allowed the rapid assembly of operons and genes clusters for co-expression of heterologous genes, a feature that may assist metabolic pathway engineering.

**Conclusions:**

We anticipate that the pTGR platform will contribute to explore the potential of novel parts to regulate gene expression, and to facilitate the assembly of genetic circuits for metabolic engineering of *C. glutamicum.* The standardization provided by this approach may provide a means to improve the productivity of biosynthetic pathways in microbial factories for the production of novel compounds.

## Background

Synthetic biology is an emerging discipline that aims to create novel organisms containing designed genetic circuits [1-4]. These circuits are built from standard biological parts, known as BioBricks, that in most of the cases are provided by nature. The advent of genome sequencing data and cost-effective custom DNA synthesis has resulted in a significant increase in the availability of these parts and unnatural variants of them including promoters, ribosome binding sites (RBS), transcriptional terminators, etc. In one of its many applications, synthetic biology is making a tremendous contribution to the advance of metabolic engineering approaches by reducing the development time of engineered organisms as a result of using parts that provide predictable response [5,6]. Thus, the progress made in this field may enable the design and construction of microbial cell factories for the production of novel chemical compounds and the improvement of the economics of existing processes by increasing the yields of desired compounds as a result of using engineered producing microorganisms.

So far, the vast majority of synthetic biology tools have been developed for *Escherichia coli*[7]. Our laboratory aims to extend the principles by designing approaches for rapid engineering of other species for which available engineering tools are scarce. For this purpose, we have undertaken an endeavor to develop strategies to: (i) rapidly identify and characterize parts involved in gene expression regulation from microorganisms of biotechnological interest and (ii) create tools for the facile assembly of genetic constructs to manipulate pathways in such microorganisms. Our approach is to use a plasmid, where all parts are flanked by a standard set of restriction sites enabling the rapid testing of regulatory sequences. The approach was validated for *C. glutamicum*, originally used for the industrial production of L-glutamic acid, and gradually developed into an efficient producer of various compounds ranging from metabolites to recombinant enzymes [8-12]. The construction of high-producing *C. glutamicum* strains in the last decade is mostly supported by metabolic engineering efforts based on the knowledge gained from the elucidation of synthesis pathways [13]. These approaches often require simultaneous and balanced control of several genes involved in a pathway. Therefore, a toolbox including regulatory sequences (parts) to facilitate predictable gene expression is desirable.

In engineered microorganisms, co-expression of multiple genes can be achieved from synthetic operons where all the genes are expressed simultaneously. In some cases, gene products are not required at the same time or their co-expression is inconvenient because they catalyze incompatible reactions. The expression of these genes can be temporally separated by using gene clusters containing more than one transcriptional unit, where each gene or group of genes is under the control of an independent promoter from which transcription can be induced when necessary. The use of multiple transcriptional units may also facilitate the modulation of the expression of groups of genes by placing them under the control of promoters with different strengths.

To date, only a limited set of well characterized vectors are available for *C. glutamiucum*[14-17]. The design of platforms for exploring the potential of novel parts to regulate gene expression and for the assembly of genetic circuits for metabolic engineering may allow for the improvement of the capacity of this biotechnology workhorse as a producer of valuable compounds with the consequent economic impact.

Here we provide a synthetic biology platform for the rapid evaluation of sequences to regulate gene expression and for the assembly of polycistronic operons and clusters containing multiple transcriptional units in *C. glutamicum*.

## Results

### Vector features and construction

**Figure 1:**
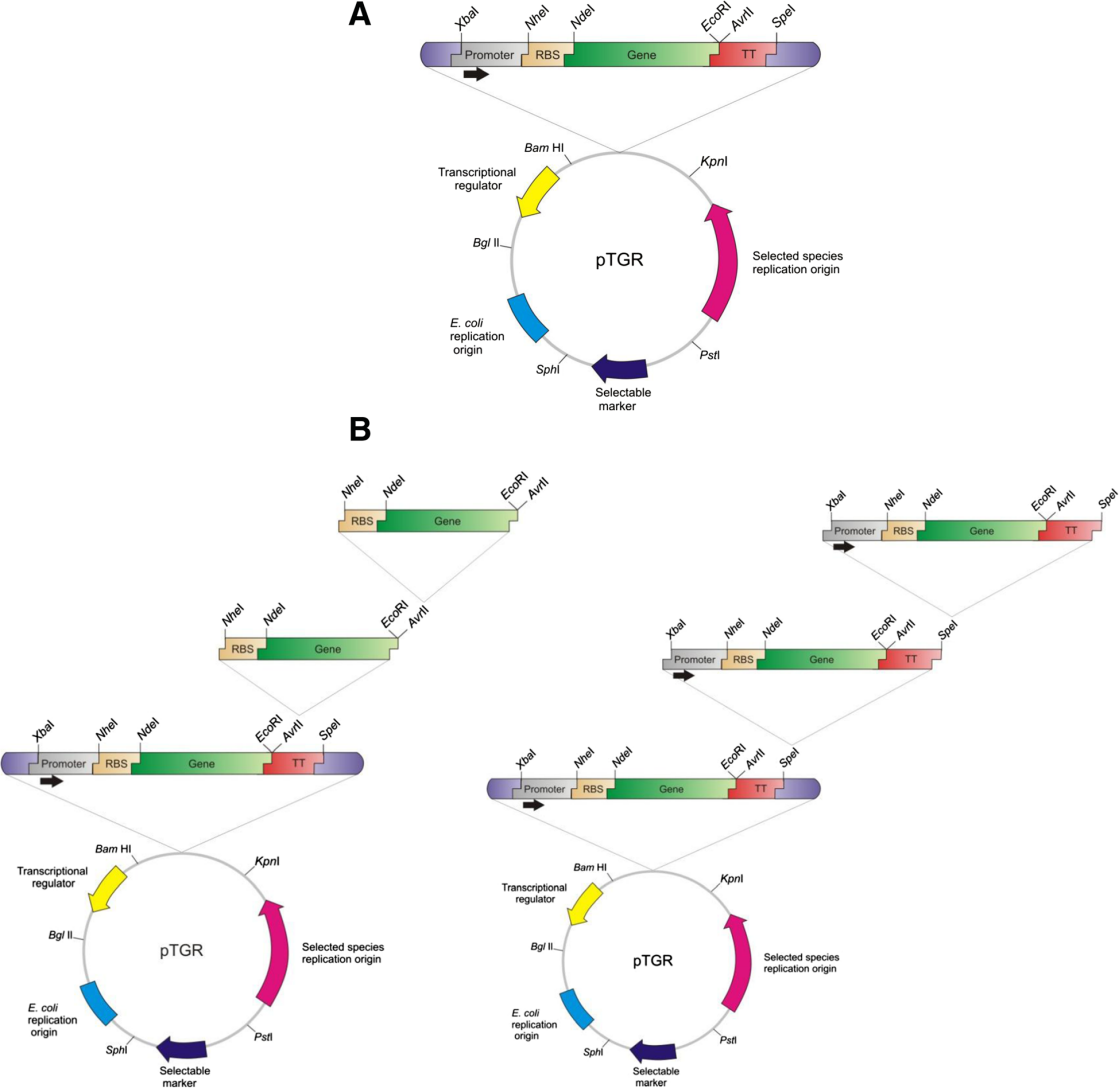
**pTGR platform features.** (**A**) Map of the generic pTGR synthetic plasmid where all parts are flanked by a standard set of unique restriction sites. (**B**) The two types of constructions for the expression of multiple genes. Sequential insertion of cassettes containing *RBS-ORF* for the assembly of operons or cassettes comprising *Promoter-RBS-ORF*-*transcriptional terminator* for the assembly of gene clusters.

The series of synthetic plasmids was named pTGR, the generic vector is illustrated in Figure 1A and the all the derivatives used in this study are listed in Table 1 and a schematic representation is shown in Additional file 1. The plasmid posses the following eight parts: (i) a replication origin for the host, (ii) a replication origin for *E. coli*, (iii) a selectable marker, and a transcriptional unit cassette containing: (iv) a transcriptional regulator, (v) a promoter with an corresponding operator, (vi) a RBS, (vii) the gene to be expressed and (viii) a transcriptional terminator.

**Table 1:** The pTGR series of plasmids used in this study

**Plasmid**	**Promoter**	**RBS**	**Gene**	**Replicon**
pTGR1	*-*	*-*	eGFP	pGA1 mini replicon
pTGR2	*-*	*sod*	eGFP	pGA1 mini replicon
pTGR3	*sod*	*sod*	eGFP	pGA1 mini replicon
pTGR4	*csp*B	*sod*	eGFP	pGA1 mini replicon
pTGR5	*tac*	*sod*	eGFP	pGA1 mini replicon
pTGR6	*tac*	*lacZ*	eGFP	pGA1 mini replicon
pTGR7	*tac*	*csp*B	eGFP	pGA1 mini replicon
pTGR8	*tac*	*sod* / *sod*	mCherry /eGFP	pGA1 mini replicon
pTGR9	*tac* / *sod*	*sod* / *sod*	eGFP/ mCherry	pGA1 mini replicon
pTGR10	*tac*	*lacZ* / *sod*	mCherry /eGFP	pGA1 mini replicon
pTGR11	*tac*	*lacZ* / *lacZ*	mCherry /eGFP	pGA1 mini replicon
pTGR12	*tac*	*sod* / *lacZ*	mCherry /eGFP	pGA1 mini replicon
pTGR13	*sod* / *tac*	*sod* / *sod*	eGFP/ mCherry	pGA1 mini replicon
pTGR14	*tac* / *cspB*	*sod* / *sod*	eGFP/ mCherry	pGA1 mini replicon
pTGR15	*cspB* / *tac*	*sod* / *sod*	eGFP/ mCherry	pGA1 mini replicon
pTGR16	*tac*	*sod*	eGFP	pNG2 minimal replicon
pTGR17	*tac*	*sod*	eGFP	pCRY4 minimal replicon

In all the cases the ColE1 origin of replication from *E. coli*, and the *rrnBT1* and *rrBT2* in tandem transcriptional terminators, the Tn5 kanamycin resistance marker and a cassette for the expression of the LacI repressor were used.

Each component of the plasmid is flanked by unique restriction sites for easy exchange of all parts. The *E. coli* origin of replication is flanked by BglII and SphI sites, the selectable marker by SphI and PstI, and the replication origin for the selected host by PstI and KpnI. BamHI and BglII permit the insertion of genes encoding transcriptional regulators. In this plasmid, the cassettes for both the selectable marker and the transcriptional regulator must contain the necessary regulatory sequences for expression.

The transcriptional unit fragment was designed with XbaI and SpeI sites at the 5' and 3' borders respectively. Within this cassette, the promoter/operator part is limited by XbaI and NheI, the RBS is flanked by NheI and NdeI, the genes to be expressed are always cloned between NdeI and EcoRI and the transcriptional terminator is placed between AvrII and SpeI. The design is such that multiple genes, like for example those encoding the enzymes of an entire pathway, can be expressed from this plasmid at different levels by using promoters with variable strengths or RBS with different affinities. Constructions for the simultaneous expression of various genes can be rapidly obtained in two different formats, by assembling operons or clusters with multiple transcriptional units.

As shown in Figure 1B, operons can be assembled by cloning each gene into the NdeI-EcoRI sites, excising the NheI-AvrII fragment of one construction and inserting such fragment into the AvrII site of the second plasmid. NheI and AvrII digestion results in compatible cohesive ends. Thus, insertions in the correct orientation generates an NheI/AvrII scar separating the stop codon of the first gene and the RBS of the second gene and the AvrII site downstream the stop codon of the second gene is regenerated, allowing its recursive use to add further genes to the operon. Similarly, clusters containing multiple transcriptional units, with operons or single genes, can be assembled by using the XbaI and SpeI sites. In this case, one cluster is excised with XbaI and SpeI and inserted into the SpeI site of the acceptor plasmid containing a second cluster. The resulting vector has an XbaI/SpeI scar at the joint of the two clusters and the SpeI site is regenerated and available for the insertion of a new cluster.

### Testing promoter and RBS activities in *C. glutamicum* using the pTGR system

In one of its uses, the generic plasmid pTGR is expected to serve as a probe vector for the rapid evaluation of promoters and RBSs in order to obtain a collection of these parts to facilitate tunable gene expression in metabolic engineering approaches. Thus, to experimentally validate our strategy, the synthetic plasmid pTGR1 containing the mini replicon from the *C. glutamicum* pGA1 plasmid [18], the ColE1 origin of replication from *E. coli*, and the *rrnBT1* and *rrBT2* in tandem transcriptional terminators was created where all the parts are flanked by the above described restriction sites. Next, a synthetic version of a gene encoding an enhanced green fluorescence protein (eGFP) [19] was inserted into the NdeI-EcoRI sites to be used as a reporter; and the RBS from *C. glutamicum sod* gene inserted between the NheI-NdeI sites of the resulting plasmid to create the pTGR2. Three synthetic DNA fragments containing promoter sequences from the *sod* and *cspB* genes from *C. glutamicum*, and the *E. coli* hybrid *tac* promoter were cloned into the XbaI-NheI sites of the pTGR2 to obtain the pTGR3, pTGR4 and pTGR5 vectors respectively. In the plasmid containing the *tac* promoter, a synthetic cassette for the expression of the LacI repressor was inserted between the BamHI-BglII sites. These promoter sequences were chosen based on activities reported by other authors in heterologous gene expression experiments in *C. glutamicum*[20-22].

**Figure 2:**
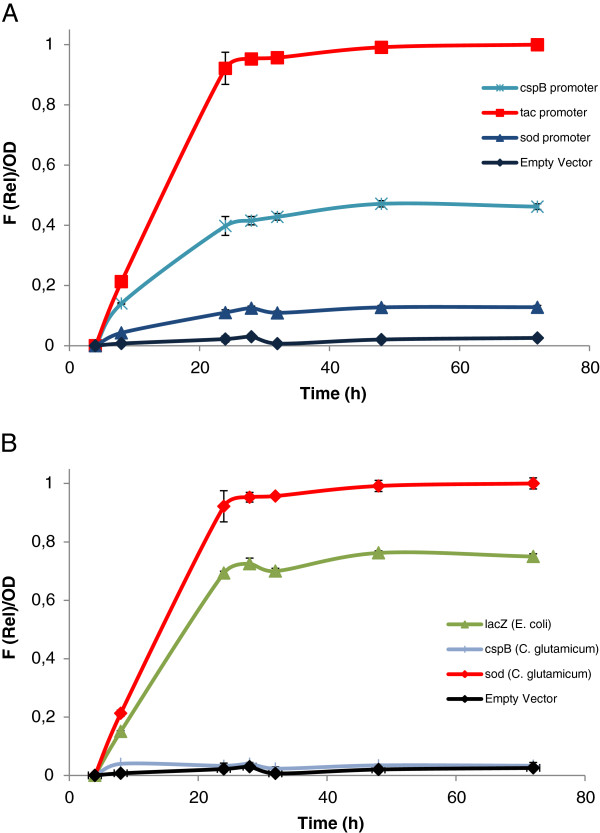
**Assessment of promoter and RBS activities in C. glutamicum with the pTGR system.** (**A**) Fluorescence intensity of eGFP relative to OD during the growth of *C. glutamicum* using different promoters to drive the expression of the reporter gene. (**B**) Fluorescence intensity of eGFP relative to OD testing three different RBSs. Cultures were grown in BHIS medium and supplemented with 0.5 mM IPTG when indicated. Values shown are means of three independent determinations. The standard deviations were in all the cases less than 10% of the corresponding means.

Figure 2A shows eGFP expression under the control of three different promoters in *C. glutamicum* ATCC 13869 cultivated in BHIS medium. In experiments where the expression was driven by the *tac* promoter, 0.5 mM IPTG was added to the cultures. The expression from *sod* and *cspB* does not require the addition of an exogenous inducer since these promoters are growth phase dependant [23]. A similar rate of induction was observed for the three promoters, and the maximum amount of eGFP per cell was obtained after 24 hours; when cultures reached stationary phase. Background expression of eGFP was not detected in the absence of promoter. Clearly, the three promoters used to test the pTGR system provide different levels of expression under the tested conditions: high (*tac*), medium (*cspB*) and low (*sod*), showing that the pTGR may serve to rapidly evaluate and classify promoter parts.

To test RBS activities, the Shine-Dalgarno sequence of the pTGR3 was substituted by those from the *E. coli lacZ* gene, and the *cspB* gene to create the pTGR6 and pTGR7 plasmids respectively. The distance between the Shine-Dalgarno sequence and the ATG start codon of the native genes, a critical factor affecting translation, was preserved in all the cases. The plasmids pTGR3, pTGR6 and pTGR7, all of them containing the eGFP under the control of the *tac* promoter but with three different RBS sequences, were transformed into *C. glutamicum*. Cultures were grown on BHIS medium supplemented with IPTG, and fluorescence/OD ratio was determined. Figure 2B shows that eGFP expression can be modulated by exchanging RBSs. The *sod* RBS provided the highest expression level, the *lacZ* RBS provided an intermediate amount of eGFP while the expression using *cspB* RBS was barely higher than the negative control. The result of this study indicates that the pTGR system can be used to identify, test and classify new RBS parts by means of a simple fluorometric assay.

### Testing operon and gene cluster constructions in *C. glutamicum*

**Figure 3:**
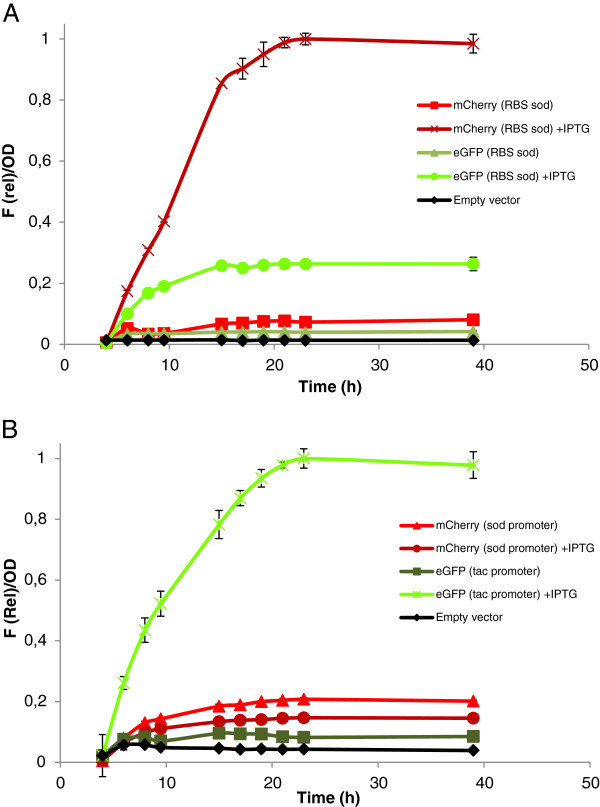
**Evaluation of the co-expression of two reporter genes from pTGR constructs.** (**A**) Time course of the co-expression of eGFP and mCherry genes contained in an operon relative to OD. (**B**) Time course of the co-expression of eGFP and mCherry genes contained in two transcriptional units relative to OD. Cultures were grown in BHIS medium and supplemented with 0.5 mM IPTG when indicated. Values shown are means of three independent determinations. The standard deviations were in all the cases less than 10% of the corresponding means.

The pTGR system was designed to facilitate constructions for the expression of multiple genes. For this, two possible formats are possible: gene assembly into operons, or clusters containing more than one transcriptional unit. In order to validate the system, an operon was initially created with two reporter genes, one encoding for eGFP and the second for mCherry fluorescent protein [24]. The operon was constructed in just two cloning steps to obtain the pTGR8 plasmid. First the synthetic ORFs encoding both fluorescent proteins were inserted into the NdeI-EcoRI sites of a pTGR vector containing the *tac* promoter and the *sod* RBS and second, the NheI-AvrII fragment containing *sod* RBS-eGFP ORF was mobilized to the AvrII site of the plasmid containing the mCherry gene. Expression of the resulting operon successfully co-produced the two fluorescent proteins (Figure 3A).

Next, a cluster containing two transcriptional units was constructed to validate the second class of assembly strategy. This construction also required two cloning steps. First the synthetic ORFs encoding the two fluorescent proteins were inserted as above into the pTGR vector containing either the *tac* or *sod* promoter upstream the *sod* RBS. Second, the XbaI-SpeI fragment containing *sod promoter-sod RBS-mCherry ORF-transcriptional terminator* was mobilized to the SpeI site of the plasmid containing the eGFP gene under the transcriptional control of the *tac* promoter, to obtain the pTGR9 plasmid. Upon IPTG induction, expression of both fluorescent proteins was obtained as shown in Figure 3B.

**Figure 4:**
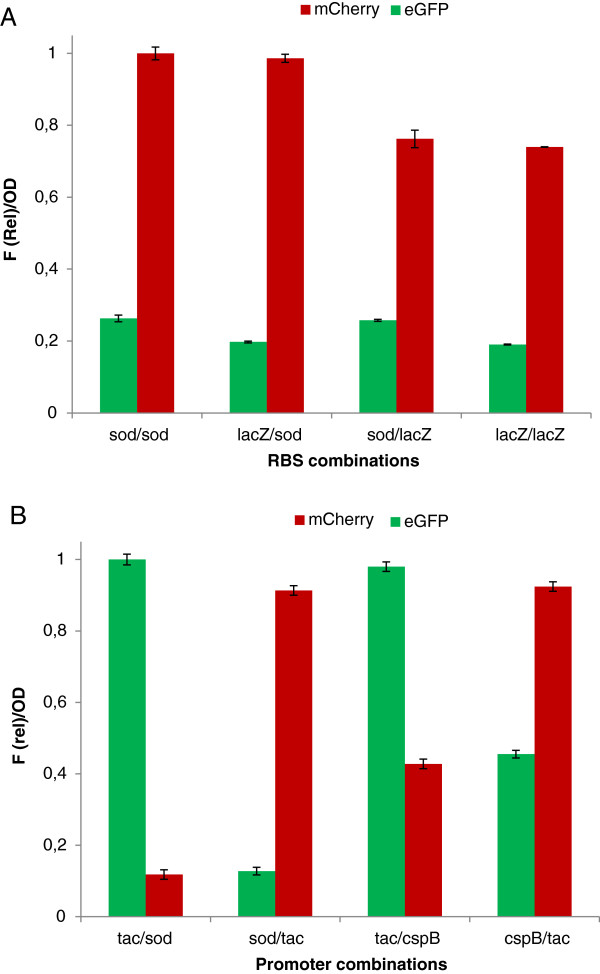
**Comparison of the co-expression levels of eGFP and mCherry genes in C. glutamicum.** (**A**) the genes were assembled in operons with the indicated RBSs controlling the expression. In all cases operons are transcribed from the *tac* promoter. (**B**) the genes were co-expressed from two transcriptional units and the indicated promoter drives the expression of each gene. Fluorescence intensity of eGFP and mCherry relative to OD after 24 h of incubation of cultures is shown. Cultures were grown in BHIS medium and supplemented with 0.5 mM IPTG when the *tac* promoter was used. Values shown are means of three independent determinations. The standard deviations were in all the cases less than 10% of the corresponding means.

To demonstrate that the pTGR system allows the amount of both proteins to be altered by either using RBSs with different stregths in the case of operons, or promoters of different strengths in the case of multiple transcriptional units; a further set of experiments was carried out. First, the operon contained in plasmid pTGR8 was modified by exchanging the *sod* RBS that controls the translation of mCherry with the *lacZ* RBS to obtain the pTGR10 plasmid. As expected, when using this construct the amount of mCherry is lower than that obtained when the *sod* RBS is used (sod/sod and sod/LacZ, bars, Figure 4A). Next, a new plasmid designed pTGR11 was created where the *sod* RBS controlling the translation of eGFP is replaced by *lacZ* RBS, and the anticipated reduction in the expression was achieved confirming the feasibility of this approach to tune gene expression.

Finally, three additional clusters containing two transcriptional units were created where different combinations of promoters were used to drive the expression of eGFP and mCherry. Figure 4B shows that the ratio of production of the two proteins can be also altered by using promoters with different strengths, indicating that this strategy is feasible to modulate the expression of multiple genes as well.

### Tuning gene expression by varying inducer concentration and replication origin swapping

**Figure 5:**
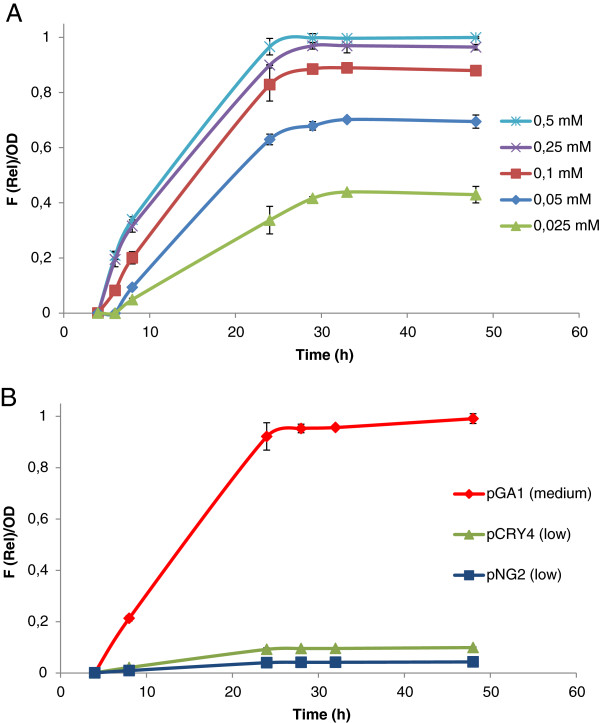
**Effect of IPTG concentration and plasmid copy number on eGFP expression in *C. glutamicum *using the pTGR system.** (**A**) Fluorescence intensity of eGFP relative to OD during the growth of *C. glutamicum* using different IPTG concentration to drive the expression of the reporter gene. (**B**) Fluorescence intensity of eGFP relative to OD testing three different replicons. pGA1 (medium copy number), pCRY4 (low copy number) and pNG2 (low copy number.) Cultures were grown in BHIS medium and supplemented with the indicated concentration of IPTG at OD_590_ = 0.5. Values shown are means of three independent determinations. The standard deviations were in all the cases less than 10% of the corresponding means.

In order to explore further alternatives to tune gene expression using the pTGR platform, the level of expression of eGFP was tested under different inducer concentrations. Using pTGR5 vector, eGFP expression driven by *tac* promoter was induced with IPTG concentrations ranging 0.025-0,5 mM. The obtained results (Figure 5A) show a variation in gene expression between 0.025-0,25 mM, and no further increase in fluorescence relative to OD was obtained above the higher concentration, suggesting a saturation effect. These results indicate that a new instance of regulation can be exploited when using the *tac* promoter to drive the expression of heterologous genes with the pTGR system.

Varying gene dose is another well establish method to regulate gene expression. To test the feasibility of this approach using the pTGR platform, the replication origin of the medium copy number plasmid pGA1 (~30 per cell) present in the pTGR5 was replaced by the low copy number origins (1–2 per cell) from pCRY4 [25] and pNG2 [26-28]. Figure 5B shows that the expected decrease in gene expression through reduction of plasmid copy number was achieved, indicating that this strategy may also be suitable to tune the expression of heterologous gene. Although this application is currently limited by the low number of replication origins described and characterized in *C. glutamicum*[29], the pTGR platform could provide a new means for a preliminary characterization of replication origins*.*

## Discussion

There is an increasing demand for the production of novel small molecules and biomaterials including drugs, chemicals, biofuels and biopolymers. To efficiently produce these molecules and to reduce the production cost of existing compounds in engineered microbial cells, a major challenge is to regulate the expression of a large number of genes to optimize the production of a biosynthesis pathway. The introduction of genes into production hosts is typically achieved using plasmids previously assembled and propagated in *E. coli*. Synthetic biology can make a significant contribution to speed up this process by using standardized plasmids where all the components are synthesized with standard formats to facilitate easy exchange and testing [3,30].

Here we present a synthetic biology framework for genetic engineering of *C. glutamicum*, a microorganism of industrial interest for which synthetic biology tools have not been developed yet. In the design described herein, all the plasmid components are flanked by unique restriction sites to enable a standardized replacement of genetic elements. In most cases, the parts and pathway genes used in our laboratory are synthetic and designed so as to avoid NdeI, EcoRI, XbaI, SpeI, NheI and AvrII sites, although natural sequences lacking these sites and PCR amplified to add the flanking restriction sites are used as well. Short regulatory sequences, like promoters and RBSs, can be simply added by using overlapping oligonucleotides. Codon usage of heterologous genes can be optimized to facilitate its expression in *C. glutamicum*. The whole process of gene design, including restriction site generation/removal and codon optimization, can be achieved in one step using free web based programs like Optimizer [31,32].

Several formats for the construction of synthetic plasmids and for the assembly of parts have been proposed [30,33-36]. The most accepted method in the synthetic biology community was created by Knight and co-workers [37]. They proposed the BioBrick standard for the assembly of biological parts, where all parts are flanked by a standard set of restriction sites to allow joining and combination with further parts. The plasmids described here can be adapted to fit the BioBrick assembly method by replacing the set of restriction sites, although some limitations have been described for this format as well [38].

Optimal levels of enzyme to maximize production from a biosynthetic pathway can be achieved as a result of fine tuning gene expression by, for example, modulating transcription or translation. For this purpose, a toolbox with a collection of promoters and RBSs capable of providing different levels of gene expression is desirable. The pTGR system may serve as a standardized test to evaluate the strength of promoter sequences. To validate this application, three promoters were tested using the eGFP as a reporter gene. Synthetic DNA fragments containing the *E. coli tac* promoter, and the *sod* and *cspB* promoter from *C. glutamicum* were inserted using the standard format. Three levels of fluorescence intensity were obtained: the *tac* promoter provided the strongest signal, the *sod* promoter the lowest amount of fluorescence and the *cspB* an intermediate intensity. The result is not surprising since, (i) other groups have used *E. coli lac* derived promoters like *tac* and *trc* to over-express genes in *C. glutamicum*[15,39,40], and (ii) the sequence of the −10 box of the *tac* promoter is identical to that of the consensus promoter of *C. glutamicum*[23]. Interestingly, this promoter may provide another instance for fine tuning gene expression by using different concentrations of the IPTG inducer [41]. Although a GFP based approach to characterize promoters has been previously described by Knoppova and co-workers [16], the vector described does not possess the versatility of the pTGR platform for testing multiple regulatory sequences.

Initiation is the rate limiting step of translation. Provided that there are not secondary structures between the RBS and the coding sequence, it was shown that RBSs can be used as a regulatory part since they affect translation initiation and, therefore, gene expression [42,43]. Moreover, a method was recently described for automatic design of artificial RBSs to control gene expression, expanding the potential of these sequences to be used in genetic circuits [44]. In this way, the pTGR may also be used to test RBSs modulator effect on gene expression. To validate this application, three different RBSs were tested using eGFP as a reporter gene under the *tac* promoter. The sequences provided variable amounts of fluorescence intensity, validating the use of the system to populate a collection of these gene-expression regulatory sequences.

The pTGR provides a rapid means to create constructions for the expression of multiple genes. The design allows the assembly of constructs -operons or gene clusters- in as many cloning steps as genes are assembled by: (i) inserting all the genes to be expressed into a pTGR vector with the desired regulatory sequences and (ii) the sequential transfer of the genes with corresponding regulator sequences to a vector containing another gene(s) to extend an operon or gene cluster as illustrated in Figure 1B.

It is tempting to expect that the output of promoters and RBSs in single gene expression experiments from probe vectors may anticipate the performance of these parts in a more complex context like operons or multiple gene clusters. For example, the level of expression of both proteins in the experiments shown in Figure 4 is similar to that expected from the individual expression of each protein (Figure 3). This would facilitate the classification of regulatory parts to accurately regulate gene expression in pathways requiring multiple proteins. However, the output of many parts may be context dependant. Thus, obtaining the optimal balance for all the proteins, specially for pathways containing a high number of genes, may require multiple tests to find the appropriate regulatory sequences. Moreover, in most cases the optimal level of expression of a given protein in a pathway is unknown, and combinatorial constructions using a variety of regulatory sequences are necessary to find the right combination [45-47]. The pTGR platform may contribute to speed up these tests by facilitating the rapid assembly of combinatorial constructs and exchange of parts involved in gene expression regulation.

Other features of the pTGR platform may provide additional levels of regulation. For example, the copy number of a gene cluster or an operon may be regulated by using origins of replication from medium or low copy number plasmids. This can be easily achieved in one cloning step using the KpnI and PstI sites flanking the replication origin. Alternatively, a sequence for the insertion of the genes into the chromosome may be inserted into this place.

In principle, the platform described here for *C. glutamicum* may be extended to other microorganisms by replacing its origin of replication by an appropriate counterpart from, for example, *Bacillus* and *Streptomyces*. Such experiments are in progress in our laboratory to validate the use of the pTGR system in other microorganisms of industrial interests.

## Conclusions

The first synthetic biology framework for genetic engineering of *C. glutamicum* has been created. The platform is based on the use of plasmids where all the components influencing gene expression are flanked by unique restriction sites to facilitate the exchange of parts and the rapid assembly of constructs for the expression of multiple genes. The application has been validated by testing promoters, ribosome binding sites and by the assembly of operons and gene cluster constructs to express reporter genes. The presented platform may facilitate metabolic engineering of *C. glutamicum* to produce valuable compounds in a cost-effective manner.

## Methods

### Strains, plasmids, and growth conditions

The bacterial strains used in this work were *E. coli* DH5α [48] and *C. glutamicum* ATCC 13869. *E. coli* was grown on LB [49] at 200 rpm and 37°C and *C. glutamicum* was grown on BHIS (brain–heart infusion agar from Difco Laboratories, with 0.5 M sorbitol) at 200 rpm and 30°C. The concentrations of kanamycin used were 50 mg L^-1^ for *E. coli* and 25 mg L^-1^ for *C. glutamicum*. The plasmids used are listed in Table 1. NCBI accession number for pTGR5 plasmid is JX559328.

### DNA preparation and PCR techniques

Restrictions enzymes, T4 ligase and DNA ladders were purchased from New England Biolabs. Plasmid DNA was prepared by using the Axygen Biosciences Axy-Prep™ Plasmid Minipreps Kit. DNA sequencing was performed at the Sequencing Facility of the University of Maine. Plasmid pTGR, including all the DNA components described in this work were synthesized by Genescript (NJ, USA). In all cases *E. coli* DH5a was used for cloning. Gene codon optimization was carried out using the software Optimizer.

### Preparation of competent cells and transformation

Preparation of competent cells and transformation of *E. coli* and *C. glutamicum* were performed according to the protocol previously described [17,50].

### Fluorescence measurements

At different times, samples of the cells grown under selective conditions (with Km), were taken. For in vivo promoters and RBSs tests, fluorescence of eGFP values were measured with a BiotekR Synergy 2 Multi-Mode Microplate Reader. For in vivo operons and multiple transcriptional unit tests, the fluorescence of eGFP and mCherry values were measured with a Agilent Technologies Varian Cary Eclipse Fluorescence Spectrophotometer, to allow for an adequate separation of the contributions from each protein. The fluorescence values of eGFP and mCherry fluorescence were normalized by dividing the actual fluorescence value measured in arbitrary units by the brightness of the corresponding protein (34 1/(mM.cm) for eGFP and 16 1/(mM.cm) for mCherry [51].

The graphics report relative fluorescence to OD ratio. Relative fluorescence was calculated as the ratio between measured fluorescence to maximum fluorescence in arbitrary units. All curves within each graph are comparable, as the measurements were performed in identical instrumental conditions. The measurements of optical density were performed at 600 nm. The culture media was used as blank in all cases.

## Competing interests

The authors declare that they have no competing interests.

## Authors’ contributions

PR carried out the experimental work, SP and HG participated in the design of the study and HGM conceived the study, designed the experiments and wrote the manuscript. All authors read and approved the final manuscript.

## Supplementary Material

Additional file 1 **Schematic representation of the constructs expressed from the pTGR series of plasmids used in this study.** The region between XbaI and SpeI sites for the pTGR plasmids containing promoters (*tac*, *sod* or *cspB*), RBSs (*lacZ, sod or cspB*), reporter gene (eGFP or mCherry) and the *rrnBT1* and *rrBT2* in tandem transcriptional terminators. (PDF 278 kb) Click here for file
