# Supplementary figures and images for: Design and testing of a synthetic biology framework for genetic engineering of Corynebacterium glutamicum
Source: Microb Cell Fact. 2012 Nov 7;11:147. doi: 10.1186/1475-2859-11-147 (PMC3539996; doi:10.1186/1475-2859-11-147)

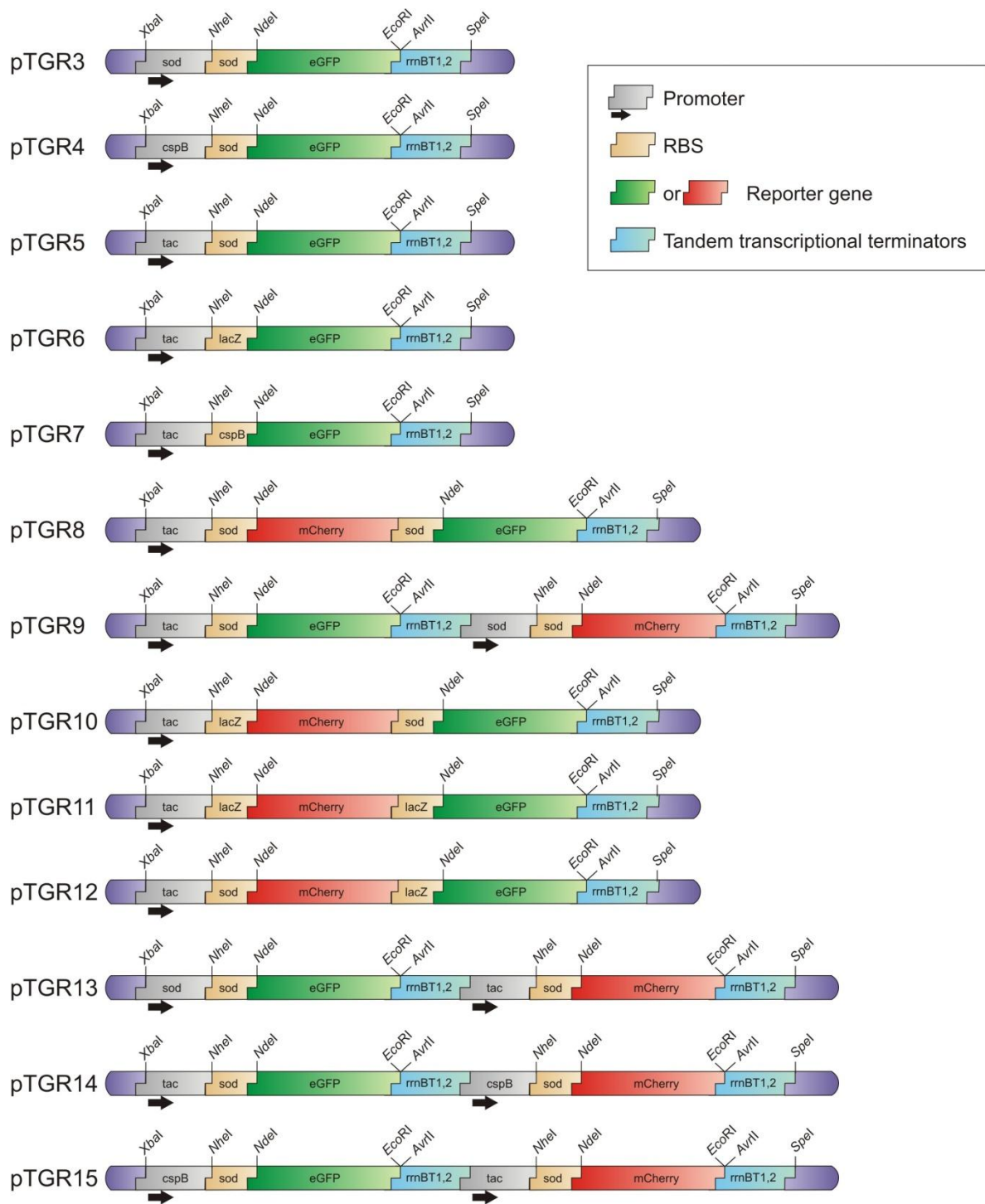

Supplementary fig. 1 Ravasi et. al

Supplement: Additional file 1 — Schematic representation of the constructs expressed from the pTGR series of plasmids used in this study. The region between XbaI and SpeI sites for the pTGR plasmids containing promoters (tac, sod or cspB), RBSs (lacZ, sod or cspB), reporter gene (eGFP or mCherry) and the rrnBT1 and rrBT2 in tandem transcriptional terminators. (PDF 278 kb) [file 1475-2859-11-147-S1.pdf]
